# Research on low-carbon dual channel supply chain considering product substitution under government carbon tax and low-carbon subsidy

**DOI:** 10.1371/journal.pone.0287167

**Published:** 2023-06-16

**Authors:** Changyan Xu, Xin Tang, Jingyao Song, Chuanxu Wang

**Affiliations:** 1 School of Economics & Management, Shanghai Maritime University, Shanghai, China; 2 College of Foreign Languages, Shanghai Maritime University, Shanghai, China; Bond University, AUSTRALIA

## Abstract

Since dual channel supply chain has become one of the main modes of supply chain, its research has acquired great significance. This paper constructs a low-carbon dual channel supply chain composed of one manufacturer and one retailer. The manufacturer produces low-carbon product and high carbon product with substitution relationship. The retailer sells high carbon product in traditional channel. The manufacturer also sells low-carbon product in direct channel. The government, manufacturer and retailer conduct a three-level Stackelberg game. This paper studies the optimal decisions of the government, manufacturer and retailer under the three modes of carbon tax + subsidy, carbon tax only and subsidy only. It has been found that for social welfare, the carbon tax + subsidy model is higher than the subsidy model and carbon tax model. For manufacturer profit, the subsidy mode is the highest, followed by the carbon tax + subsidy mode. For retailer profit, the carbon tax + subsidy model is equal to the carbon tax model. The increase in the proportion of consumers who prefer high carbon product in the total market or product cost of low-carbon product, will increase the profit of traditional channel and reduce the profit of direct channel.

## 1. Introduction

In the past two decades, the emission reduction of greenhouse gases, mainly represented by carbon dioxide, has become an increasingly concerned issue globally [1]. The abnormal climate events all over the world in recent years have attracted people’s attention to the global warming phenomenon. The concept of low carbon has increasingly infiltrated into all aspects of society, especially in the economic field ([2, 3]). As a major carbon emission country, China has put forward specific implementation plans such as "Blue Sky Action", made efforts in the emission of air pollutants and greenhouse gases, and made significant contributions to the improvement of the global atmospheric environment. In 2019, China further solemnly made a commitment to the "double carbon" goal to the world [4]. The construction and management of low carbon supply chain is an important component and necessary means of low carbon economy development. The core of low carbon supply chain is to replace high carbon product with low carbon product. It requires cooperative efforts from manufacturers to retailers, and it is a long term and gradual process, because consumers also have their own buying habits and preferences.

With the rapid development of e-commerce, many enterprises choose the dual channel, such as Nike, Dell and Apple [5]. Enterprises can reach more consumers through direct channel to achieve the goal of rapid product promotion. In practice, many manufacturers will choose traditional channel to sell products without technological innovation and direct channel sell products with new technologies to consumers [6]. Traditional channel product and direct channel product usually have a certain degree of substitutability in terms of functionality, therefore the two products have market competition. It is very complicated for manufacturer to determine the channel strategy, as well as the selling prices of the two products. Manufacturers and retailers in the dual channel supply chain should take into account how to efficiently realize the comprehensive construction of the low carbon dual channel supply chain, and formulate reasonable wholesale and retail prices, coordinate sales channels for low carbon product and high carbon product. These are not only the heated issues in the current academic areas, but also the problem that practitioners of all the industrial sections should consider and solve [7]. The development of low-carbon economy, especially the low carbon dual channel supply chain, should first be regulated by the government which plays a leading role, formulates corresponding laws and regulations, integrates and allocates social resources, as well as supports the development of low-carbon industry and the promotion of low-carbon technology [8]. Government policies that have a direct impact on the low carbon supply chain mainly include carbon tax and low carbon subsidy. At present, carbon tax is chiefly levied in some developed countries and regions, mostly in Europe. And their experiences have proved that carbon tax levied on enterprises can reduce carbon emission and even change the consumption pattern of ordinary people, the impact of this measure on enterprises, environment and national competitiveness needs careful investigation and assessment ([9, 10]). China is also actively preparing for the establishment of a carbon tax system. The special research panel on carbon tax led by the Development and Reform Commission and the Ministry of Finance has issued a research report, which analyzes the necessity and feasibility of levying carbon tax in terms of theory, policy and technology, and puts forward the basic objectives and principles of levying carbon tax in China [11]. On the other hand, government subsidy for low-carbon supply chain can also encourage enterprises to optimize the construction of green supply chain and play an incentive role in carbon emission reduction. For example, <Interim Measures for the Administration of Subsidies for Energy Conservation and Emission reduction> (2015) issued by the Ministry of Finance of China aims to encourage enterprises to increase investment in energy conservation and emission reduction by using subsidy policies [12]. In addition, the government’s low carbon subsidy to consumers buying low carbon product will also change people’s purchase choices of products and accelerate the substitution of low carbon product [13].

Under such background, we propose the following questions:

1. How do high carbon and low carbon product compete with each other in a dual channel supply chain?
2. When the government carries out carbon tax, low-carbon subsidy or both policies at the same time, what are their differences? Which policy mode is the best for carbon emission reduction?
3. When the government carries out carbon tax, low-carbon subsidy and both, what are their influences on the decision-making of dual channel supply chain participants, i.e. manufacturers and retailers?

The remaining of the paper is arranged as the following sections: Section 2 reviews the previous research literatures, summarizes their main directions as well as shortcomings, and points out the gaps to be filled in this study. Section 3 is the introduction of the research model, which describes the dual channel supply chain model considering the substitution of high carbon and low carbon product established in this study, and the definition of the parameters in the model. Section 4 calculates the performance of the above supply chain model under the three modes of subsidy, carbon tax and subsidy + carbon tax, focused on performance of parameters such as product demand, manufacturer profit, retailer profit and social welfare. Section 5 is a numerical example analysis, bringing in specific values from real cases to further test the numerical performance of each parameter of the supply chain considering the two products under the three modes. Section 6 is the conclusion, which summarizes the main findings and significance of this study, reviews existing defects to be further improved, and makes a prospect for future research.

## 2. Literature review

To construct low-carbon supply chain and promote it to replace the traditional high-energy-consuming supply chain forms, theoretical researches should be carried out concerning all the aspects in low-carbon supply chain development. In this section we have a review about the literatures falling into three topics: low-carbon supply chain; substitution of low-carbon product; low-carbon tax and subsidy toward supply chains.

Low-carbon supply chain is first of all a management problem (LCSCM, low-carbon supply chain management). Many studies use mathematical methods such as game theory to analyze the specific strategy adjustment and possible problems of enterprises introducing low-carbon supply chain, and put forward practical suggestions ([14]). Awan [15] highlighted the sustainability performance of manufacturer firms, for which social supply chain practices are of crucial meaning. He et al. [16] theoretically proved that the strategy of bilateral participation contract is more effective in dynamic cooperation to reduce carbon emissions by using the differential game method, because it can reduce the unfair distribution among the participants in the supply chain. There are also some enlightening studies on specific links and problems in the low-carbon supply chain. Zhang et al. [17] analyzed the content and importance of cooperation in the low-carbon supply chain and the equilibrium policy by using cooperative game. It is also suggested that the government should improve its position and strengthen public participation and supervision in the construction of low-carbon supply chain. Li and Shi [18] proposed an effective scheme to measure the performance of low-carbon supply chain by using the method of combining the Balanced Scorecard and Data Envelopment Analysis. Tanimizu et al. [19] focused on the integrated production and low-carbon transportation links that play an important role in the operation of the low-carbon supply chain. Using the technical means of genetic algorithm, beam search and heuristic rule, they established a model that can minimize the carbon emissions in the production and transportation links, and verified the feasibility of the model with a simulation system. The summarizing and evaluating the development of low-carbon supply chain in specific countries had also attracted attention. Nishitani et al. [20] conducted an empirical study on 139 Japanese manufacturing companies in various fields, and proved that companies adopting low-carbon supply chain management mode perform better in carbon emission reduction. Gupta and Jayant [21] surveyed many enterprises with questionnaires, and analyzed the results with a fuzzy DEMATE based technology, summarizing the main obstacles encountered by Indian enterprises in the process of developing low-carbon supply chain. The development and expansion of low-carbon product is the main direction of the low-carbon supply chain at the manufacturer’s end. Many scholars integrate the consideration of carbon footprint into the production model to obtain a low-carbon design scheme [22]. He et al. [23] introduced the Lagrange relaxation-based method to track and control the carbon footprint of the low-carbon product during its whole life cycle. For manufacturers, cost control of low-carbon product is a key issue to be considered. The choice preference of consumers between low-carbon and high carbon product is also worthy of attention. It will affect the pricing, profit distribution and cost sharing of retailers and manufacturers [24]. The model of Liu et al. [25] is based on the premise that consumers’ preference has to a large extent turned to low-carbon product. The higher price paid for low-carbon product makes retailers willing to share the cost of carbon emission reduction with manufacturers. Therefore, this cooperation within the low-carbon supply chain benefits both parties. Zhang et al. [26] conducted a two-level game considering the manufacturer’s strategy and the consumer’s preference, and obtained the equilibrium strategy of two-stage production, proposing a scheme that the manufacturer uses low-carbon raw materials in different stages to obtain the optimal retail price profit and the minimum carbon emission. In general, the researches on low-carbon supply chain focused mainly on discussing which innovations enterprises adopt in technology and management to achieve carbon emission reduction, and how to improve specific links such as production and transportation.

Since the development of e-commerce, dual channel supply chain had become a research hotspot in the academic community ([27, 28]). Research on dual channel mainly focuses on price and profit optimization. Niu et al. [29] compared the retail price of traditional channel and dual channel. They found that in the case of lower logistics costs for online orders than traditional channel, if the manufacturer adopted a unified price for both online channel and traditional channel, the total order would decrease and the retail price would increase. Dai et al. [30] studied a dual channel supply chain decision making problem considering supply chain members in four information sharing scenarios, and analyzed the impacts of forecasting information on product quantity decisions and wholesale price decision. Ranjit et al. [31] analyzed the impact of decision maker changes on optimal prices and profits in dual supply chain, and the findings indicate that the optimal prices of the sequential game in the follower position would not exceed in the leader position. Wang et al. [32] analyzed the selling model selection problem of manufacturer in dual channel supply chain, and found that the manufacturer prefers direct selling mode when the direct operating cost redacted. Zhao et al. [33] established a dual channel supply chain consisting of multiple manufacturers and one retailer considering green promotion. The result demonstrated that green promotion could increase profits, and the increase in costs would have a greater impact on retailer profit than manufacturer profit. Huang et al. [34] studied the impact of countervailing incentives on dual channel supply chain decision making under asymmetric selling cost information. The research showed that, when the selling cost decrease, information asymmetry may affect channel efficiency in the dual-channel. We can see that, there are not many papers on dual channel supply chain considering multiple products with substitution relationship, especially related to the government’s low carbon policies.

The government has many policy means to promote the development of low carbon economy, such as tax cap, tax trade market, etc., among which carbon tax is a policy tool that has been used by some countries and has the advantage of low implementation cost. The carbon tax levied by the long supply chain network has a great impact on the production, transportation and sales of the supply chain. How to reconstruct the supply chain in the context of carbon tax and obtain the optimal benefits is a topic that needs the attention of the academic community. Alnourani and mejjaouli [35] proved that the carbon tax has changed the behavior of the participants in the two level supply chain, affecting the selection of suppliers and the total cost, and suggested that the government consider the effect of the carbon tax on the supply chain network. Meng et al. [36] discussed the optimal strategy of product selection between two companies with competitive relationship under the conditions of Nash game and Stackelberg game respectively under different carbon tax rates. It was found that the two companies would choose the same product strategy in the Nash game and have nothing to do with carbon tax rates. In the Stackelberg game, however, they would adopt different product strategies on low carbon tax rates. Yang et al. [37] quantified and compared the impact of three different types of carbon tax policies on the supply chain network including multiple manufacturers and multiple demand markets, and obtained the differences of different tax rates in product trading volume, carbon tax and profits, as well as the impact on the decisions of supply chain participants and carbon emission reduction behaviors. In order to stimulate the development of low carbon supply chain, it is also a common policy means for the government to directly subsidize the participants and specific links. Zhang and Yu [38] predicted from the altruistic perspective what behaviors of the manufacturer as the dominant party and the retailer as the subordinate party would do to realize carbon emission reduction and what influence that would bring to their profits when the government provided compound subsidies to the low carbon supply chain. They believed that the unilateral altruistic model of the manufacturer would bring the best results in social welfare and other aspects. Ma et al. [39] constructed the game characteristics and equilibrium policies of the three channel supply chain model under the background of carbon subsidy, and found that the supply chain system dominated by manufacturers was the most stable when the government implemented the duel carbon subsidy policy. Therefore, the government should consider the power structure of the supply chain market when determining a reasonable carbon subsidy rate. From the above researches we can summarize that carbon tax and subsidy are both powerful carbon emission reduction policy tools. However, the comparison among different policy tools and the joint use of two or more policy tools, of whether they will have the same or different impacts on the profits of manufacturers and retailers in the dual channel supply chain, as well as on the social welfare generated, and what role will each policy mode play in the selection of high carbon and low carbon product, are all subjects worthy of comparative study.

To sum up, the existing researches mainly focused on the management and decision making of all participants and links in the development of low carbon dual channel supply chain, especially on the design of low carbon product from the perspective of innovation and reformation. Some studies also introduced the factors that the government imposes carbon tax on high carbon product or subsidizes low carbon product to calculate their impact on the decision making among the dual channel supply chain. However, for the relationship between low carbon product and high carbon product that are substitutive with each other in terms of demand and profit, as well as the similarities and differences in the results brought about by the government’s low carbon subsidy and carbon tax measures, and even the effects of the two joint implemented, there is still a lack of research on integrating the above factors into the same model for calculation.

## 3. Model description

This study establishes a low carbon dual channel supply chain, including a manufacturer and a retailer, in which the manufacturer produces low carbon product and high carbon product at the same time. These two products have strong substitutability in their usable functions. In recent years, with the rapid development of e-commerce, online direct channels are increasingly favored by the market, and the proportion of direct sales of manufacturers’ products in the total sales is also increasing, which has an important impact on the traditional supply chain sales channels. Traditional high carbon product mostly chooses traditional channel, while low carbon product is a new phenomenon, which is suitable for using direct channels. In terms of high carbon product, the manufacturer and the retailer play a Stackelberg game, and the manufacturer is the leader. In terms of low carbon product, the manufacturer and the retailer are engaged in Nash competition, while the government is the leader in the Stackelberg game between manufacturer and retailer. In order to encourage consumers to purchase low carbon product, the government will provide subsidy to consumers who purchase low carbon product. At the same time, the government will impose carbon tax on manufacturer of high carbon product to reduce the product of high carbon product [40].

Setting that the total market scale of low carbon product and high carbon product is *a*, and the proportion of consumers who prefer high carbon product in the total market is *h*. The production cost of low carbon product is ε times that of high carbon product. The carbon emission of low carbon product is 1/*r* of high carbon product. The parameters in the model are shown in Table 1:

**Table 1:** Parameter definitions.

Parameters	Definitions
*q*_*i*_	Market demand of product *i*, *i*= 1,2
*p*_*i*_	Retail price of product *i*
*s*	Subsidy amount for purchasing unit low-carbon product 2
*τ*	Carbon tax levied on high carbon product 1
*w*_1_	Unit wholesale price of high carbon product 1
*v*_*i*_	Production cost of unit product 1, *v*_2_ = ε*v*_1_, ε>1
*m*_*i*_	Environmental hazard caused by unit high carbon product 1, *m*_1_ = *rm*_2_, *r*>1
*h*	The proportion of consumers who prefer high carbon product in the total market
*π*_*r*_	The retailer profit
*π*_*f*_	The manufacturer profit
*π*_*g*_	The social welfare

Since consumers who buy low carbon product can directly receive subsidy from the government, it is equivalent to that the retail price of low carbon product 2 decreases from *p*_2_ to *p*_2_−*s*. Then the relationships between demand and price of high carbon product 1 and low carbon product 2 are expressed respectively as follows:

$$q_{1}=a_{1}-b_{1}p_{1}+c(p_{2}-s)$$ (1)

$$q_{2}=a_{2}-b_{2}(p_{2}-s)+cp_{1}$$ (2)

Above we have *b*_*i*_>*c*>0, *i* = 1, 2, among which *b*_*i*_>*c* indicates that the price of product *i* has a higher impact on the demand of product *i* than the substitutive product has.

## 4. Dual channel supply chain decision model analysis under three modes

### 4.1 Decision of dual channel supply chain under carbon tax + subsidy mode

**Fig 1:**
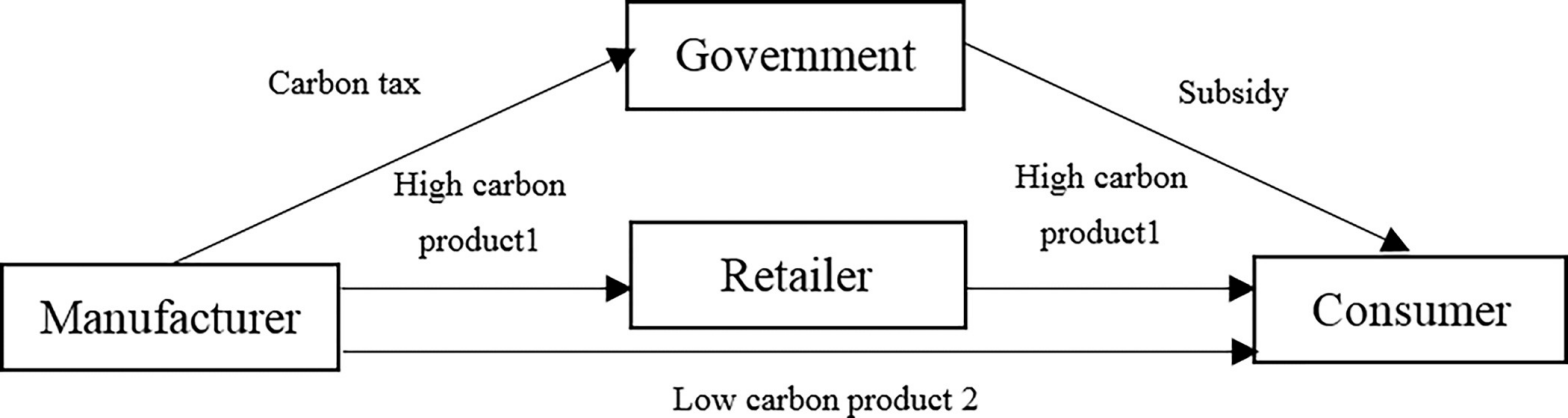
Dual channel supply chain under carbon tax + subsidy mode.

In the dual channel supply chain, we assume that high carbon product is sold by the retailer and low carbon product is directly sold by the manufacturer. The dual channel supply chain structure under carbon tax + subsidy mode is shown in Fig 1.

The retailer’s revenue is the sale income from high carbon product. The cost is the wholesale cost of high carbon product. Therefore, the retailer profit function can be expressed as:

$$Max\;\pi_{r}^{1}=q_{1}p_{1}-q_{1}w_{1}$$ (3)

*q*_1_*p*_1_ is the income received from the sale of high carbon product 1. *q*_1_*w*_1_ is the wholesale cost of high carbon product 1. Through $\frac{D\pi_{r}^{1}}{Dp_{1}}=0$ we get:

$$p_{1}^{1*}=\frac{ah-cs+cp_{2}+b_{1}w_{1}}{2b_{1}}$$ (4)

For the manufacturer, the revenue is the total wholesale income of high carbon product 1 and low carbon product 2. The cost is the total production cost of the two products plus the carbon tax levied by the government on the manufacturer to produce high carbon product 1. Therefore, the profit function of the manufacturer is expressed as follow:

$$Max\;\pi_{f}^{1}=q_{1}(w_{1}-v_{1}-\tau )+q_{2}(p_{2}-v_{2})$$ (5)

*q*_1_(*w*_1_−*v*_1_−*τ*) is the income received from wholesale of high carbon product 1. *q*_2_(*p*_2_−*v*_2_) is the income from direct channel of low carbon product 2. Through $\frac{\partial \pi_{f}^{1}}{\partial w_{1}}=0$ and $\frac{\partial \pi_{f}^{1}}{\partial p_{2}}=0$, we can acquire the optimal wholesale price of high carbon product 1 and retail price of low carbon product 2:

$$w_{1}^{1*}=\frac{b_{2}[ah+b_{1}(\tau +v_{1})]-c[a(1-h)-c\tau -cv_{1}]}{2(b_{1}b_{2}-c^{2})}$$ (6)

$$p_{2}^{1*}=\frac{c(ah-cs-c\varepsilon v_{1})-b_{1}[b_{2}(s+\varepsilon v_{1})+a(1-h)]}{2(b_{1}b_{2}-c^{2})}$$ (7)

**Theorem 1:** Subsidy and carbon tax can both increase the demand of low carbon product and reduce the demand of high carbon product.

**Prove**: According to (4) and (7), we can get that $q_{1}=\frac{ah-cs+c\varepsilon v_{1}-b_{1}(\tau +v_{1})}{4}$, and $q_{2}=\frac{c(ah-cs+c\varepsilon v_{1})+b_{1}[2a(1-h)+c\tau +cv_{1}+2b_{2}(s-\varepsilon v_{1})]}{4b_{1}}$. Since $\frac{Dq_{1}}{Ds}=-\frac{c}{4}<0$ and $\frac{Dq_{1}}{D\tau }=-\frac{b_{1}}{4}<0$, as well as $\frac{Dq_{2}}{Ds}=\frac{2b_{1}b_{2}-c^{2}}{4b_{1}}>0$ and $\frac{Dq_{2}}{D\tau }=\frac{c}{4}>0$, whether the government collects carbon tax on manufacturer or gives subsidy to consumers, both can increase the demand for low carbon product 2 and reduce the demand for high carbon product 1, and this is the same as the effect of a single channel [42].

At the same time, $\left|\frac{Dq_{1}}{Ds}\right|<\left|\frac{Dq_{1}}{D\tau }\right|$, and $\frac{Dq_{2}}{Ds}-\frac{Dq_{2}}{D\tau }=\frac{2b_{1}b_{2}-c^{2}-cb_{1}}{4b_{1}}>0$, which means that the impact of carbon tax on reducing the demand for high carbon product 1 is more obvious, while the impact of subsidy to consumers on increasing the demand for low carbon product 2 is more obvious.

The government’s goal is to maximize social welfare [41], which includes consumer surplus, retailer profit, manufacturer profit, government expenditure, government revenue and environmental hazard caused by the two kinds of products. Among them, the government expenditure is the carbon subsidy to consumers, and the government revenue is the carbon tax levied on manufacturer. Therefore, social welfare can be expressed as:

$$Max\;\pi_{g}^{1}=\psi^{1}+\pi_{r}^{1}+\pi_{f}^{1}-q_{2}s+q_{1}\tau -\sum_{i=1}^{2}q_{i}m_{i}$$ (8)

*ψ*^1^ indicates the consumer surplus, $\pi_{r}^{1}$ is Retailer profit, $\pi_{f}^{1}$ is manufacturer profit, *q*_2_*s* is the subsidy issued, *q*_2_*τ* is the carbon tax levied, and $\sum_{i=1}^{2}q_{i}m_{i}$ is the environmental hazard of two products. According to the method of Xu et al [42], setting $p_{1}=\alpha_{1}-\beta_{1}q_{1}-\gamma q_{2}.p_{2}=\alpha_{2}-\gamma q_{1}-\beta_{2}q_{2}$. Then $\psi^{1}=Τ(q_{1},q_{2})-\sum_{i=1}^{2}p_{i}q_{i}.\;Τ(q_{1},q_{2})=\alpha_{1}q_{1}+\alpha_{2}q_{2}-\frac{\beta_{1}q_{1}^{2}+2\gamma q_{1}q_{2}+\beta_{2}q_{2}^{2}}{2}$. Through $\frac{\partial \pi_{g}^{1}}{\partial s}=0$ and $\frac{\partial \pi_{g}^{1}}{\partial \tau }=0$, the optimal solution of $\pi_{g}^{1}$ can be acquired. The amount of carbon tax for unit high carbon product 1 and the amount of subsidy for unit purchase of low carbon product 2 are:

$$s^{1*}=\frac{c(ah+c\varepsilon v_{1}+2cm_{2})+b_{1}[a(1-h)-b_{2}(\varepsilon v_{1}+2m_{2})]}{b_{1}b_{2}-c^{2}}$$ (9)

$$\tau^{1*}=\frac{\begin{array}{c} 2c^{2}(ah+c\varepsilon v_{1}+cm_{2})+b_{1}^{2}b_{2}(3v_{1}+4rm_{2})-\\ b_{1}\{b_{2}(3ah+2c\varepsilon v_{1}+2cm_{2})+c[a(1-h)+3cv_{1}+4crm_{2}]\} \end{array}}{b_{1}(b_{1}b_{2}-c^{2})}$$ (10)

So we can get the optimal wholesale price of manufacturer high carbon product 1:

$$w_{1}^{1*}=\frac{(2b_{1}-c\varepsilon )v_{1}+(2rb_{1}-c)m_{2}-ah}{b_{1}}$$ (11)

And the optimal retail price of high carbon product 1 and low carbon product 2 are:

$$p_{1}^{1*}=v_{1}+rm_{2}$$ (12)

$$p_{2}^{1*}=\frac{c(ah+cm_{2})+b_{1}[a(1-h)-b_{2}m_{2}]}{b_{1}b_{2}-c^{2}}$$ (13)

The demands of high carbon product 1 and low carbon product 2 are:

$$q_{1}^{1*}=ah+v_{1}(c\varepsilon -b_{1})+m_{2}(c-rb_{1})$$ (14)

$$q_{2}^{1*}=a(1-h)+v_{1}(c-\varepsilon b_{2})+m_{2}(cr-b_{2})$$ (15)

**Theorem 2:** *h* is negatively related to *s*^1^* and *τ*^1^*.

**Prove**: $\frac{Ds^{1*}}{Dh}=-\frac{a(b_{1}-c)}{b_{1}b_{2}-c^{2}}<0$ and $\frac{D\tau^{1*}}{Dh}=\frac{a[2c^{2}+b_{1}(c-3b_{2})]}{b_{1}(b_{1}b_{2}-c^{2})}<0$, so *h* is negatively related to s^1^* and τ^1^*.

In terms of theorem 2, the more consumers who prefer high carbon product 1 there are, the less the carbon tax per unit of high carbon product 1 and the subsidy per unit of low carbon product 2. Therefore, implementing carbon tax or subsidy policies by the government can reduce consumers’ preference for high carbon product and increase their preference for low carbon product.

**Theorem 3:** $\pi_{g}^{1}$ and $\pi_{f}^{1}$ are concave functions of *h*, while $\pi_{r}^{1}$ is an increasing function of *h*.

**Prove**: The specific expressions for $\pi_{g}^{1},\pi_{f}^{1}$ and $\pi_{r}^{1}$ can be seen in Table 2. $\frac{D^{2}\pi_{g}^{1}}{Dh^{2}}=\frac{a^{2}(b_{1}+b_{2}-2c)}{b_{1}b_{2}-c^{2}}>0$, and $\frac{D^{2}\pi_{f}^{1}}{Dh^{2}}=\frac{2a^{2}[b_{1}^{2}-c^{2}+2b_{1}(b_{2}-c)]}{b_{1}(b_{1}b_{2}-c^{2})}>0$, so $\pi_{g}^{1}$ and $\pi_{f}^{1}$ are concave functions of h. Meanwhile, $\frac{D\pi_{r}^{1}}{Dh}=\frac{2cv_{1}}{b_{1}}q_{1}^{1*}$, so $\pi_{r}^{1}$ is an increasing function of *h*.

**Table 2:** The results of three models.

	Subsidy + carbon tax	Subsidy	Carbon tax
*π*_*f*_	$\frac{A1}{b_{1}(b_{1}b_{2}-c^{2})}$	$\frac{A2}{4b_{1}{\left(4b_{1}b_{2}-3c^{2}\right)}^{2}(b_{1}b_{2}-c^{2})}$	$\frac{A3}{4b_{1}(-c^{2}+b_{1}b_{2})}$
*π*_*g*_	$\frac{B1}{2(b_{1}b_{2}-c^{2})}$	$\frac{B2}{8b_{1}(+b_{1}b_{2}-c^{2})(4b_{1}b_{2}-3c^{2})}$	$\frac{B3}{8b_{1}(b_{1}b_{2}-c^{2})}$
*π*_*r*_	$\frac{{\left[ah+(ck-b_{1})v_{1}+(c-rb_{1})z_{2}\right]}^{2}}{b_{1}}$	$\frac{D^{2}}{b_{1}{\left(4b_{1}b_{2}-3c^{2}\right)}^{2}}$	$\frac{{\left(ah+(ck-b_{1})v_{1}+(c-rb_{1})z_{2}\right)}^{2}}{b_{1}}$
*q*_1_	$ah+v_{1}(c\varepsilon -b_{1})+m_{2}(c-rb_{1})$	$\frac{E}{4b_{1}b_{2}-3c^{2}}$	$ah+v_{1}(c\varepsilon -b_{1})+m_{2}(c-rb_{1})$
*q*_2_	$a(1-h)+v_{1}(c-\varepsilon b_{2})+m_{2}(cr-b_{2})$	$\frac{F}{2b_{1}(4b_{1}b_{2}-3c^{2})}$	$\frac{\begin{array}{c} b_{1}[a(1-h)+(2c-\varepsilon b_{2})v_{1}+2crm_{2}]\\ -c(ah+c\varepsilon v_{1}+2cm_{2}) \end{array}}{2b_{1}}$
*w*_1_	$\frac{(2b_{1}-c\varepsilon )v_{1}+(2rb_{1}-c)m_{2}-ah}{b_{1}}$	$\frac{c[a(h-1)+cv_{1}]-b_{2}(ah+b_{1}v_{1})}{2(c^{2}-b_{1}b_{2})}$	$\frac{G}{2b_{1}(b_{1}b_{2}-c^{2})}$
*p*_1_	$v_{1}+rm_{2}$	$\frac{H1}{2b_{1}(b_{1}b_{2}-c^{2})(4b_{1}b_{2}-3c^{2})}$	$\frac{H2}{2b_{1}(b_{1}b_{2}-c^{2})}$
*p*_2_	$\frac{c(ah+cm_{2})+b_{1}[a(1-h)-b_{2}m_{2}]}{b_{1}b_{2}-c^{2}}$	$\frac{K}{2b_{1}(b_{1}b_{2}-c^{2})(4b_{1}b_{2}-3c^{2})}$	$\frac{c(ah-c\varepsilon v_{1})+b_{1}[a(1-h)+\varepsilon b_{2}v_{1}]}{2(b_{1}b_{2}-c^{2})}$
*τ*	$\frac{L}{b_{1}(b_{1}b_{2}-c^{2})}$	_	$\frac{3(b_{1}-c\varepsilon )v_{1}-3ah-4(c-rb_{1})m_{2}}{b_{1}}$
*s*	$\frac{\begin{array}{c} c(ah+c\varepsilon v_{1}+2cm_{2})+\\ b_{1}[a(1-h)-b_{2}(\varepsilon v_{1}+2m_{2})] \end{array}}{b_{1}b_{2}-c^{2}}$	$\frac{\begin{array}{c} c(ah+c\varepsilon v_{1}+4cm_{2})+b_{1}[4a(1-h)-\\ \left(4\varepsilon b_{2}-3c)v_{1}-(8b_{2}-4cr)m_{2}\right] \end{array}}{4b_{1}b_{2}-3c^{2}}$	_

In terms of theorem 3, when $h^{1}=\frac{b_{1}\{a+b_{2}[(1-\varepsilon )v_{1}+(r-1)m_{2}]\}-c[a+c(1-\varepsilon )v_{1}+c(r-1)m_{2}]}{a(b_{1}+b_{2}-2c)},\pi_{g}^{1}$ gets the maximum value. When $h^{2}=b_{1}^{2}\{a-b_{2}[(\varepsilon -2)v_{1}-(1-2r)m_{2}]\}+c^{3}(\varepsilon v_{1}+m_{2})-cb_{1}\{a+[c(2-\varepsilon )+\varepsilon b_{2}]v_{1}+(2cr+b_{2}-c)m_{2}\}/\{a[b_{1}^{2}-c^{2}+2b_{1}(b_{2}-c)]\},\pi_{f}^{1}$ gets the maximum value.

According to theorem 3, we know that if there are too many consumers who prefer high carbon product 1, It will increase environmental pollution. The government will increase tax on manufacturer, reducing manufacturer profit. If there are too few consumers who prefer high carbon product 1, it means that the demand off low carbon product 2 will be higher, and the government will have to pay more subsidy, and manufacturer will need to pay more product cost.

**Theorem 4:** $\pi_{g}^{1},\pi_{r}^{1}$ and $\pi_{f}^{1}$ are negatively correlated with *r*.

**Prove**: Because $\frac{D\pi_{g}^{1}}{Dr}=-m_{2}q_{1}^{1*}<0,\frac{D\pi_{f}^{1}}{Dr}=-4m_{2}q_{1}^{1*}<0$, and $\frac{D\pi_{r}^{1}}{Dr}=-4m_{2}q_{1}^{1*}<0$, it can be known that $\pi_{g}^{1},\pi_{r}^{1}$ and $\pi_{f}^{1}$ are negatively correlated with *r*.

According to theorem 4, $\frac{D\pi_{g}^{1}}{Dr}=\frac{1}{4}\frac{D\pi_{f}^{1}}{Dr}=\frac{1}{2}\frac{D\pi_{r}^{1}}{Dr}$, so the change of *r* has the greatest impact on the manufacturer profit, followed by the retailer profit, and has the smallest impact on the social welfare. That is, the larger the carbon emission per unit of high carbon product 1 is, the faster decline in manufacturer profit and the slower decline in social welfare.

### 4.2 Decision of dual channel supply chain under low carbon subsidy mode

**Fig 2:**
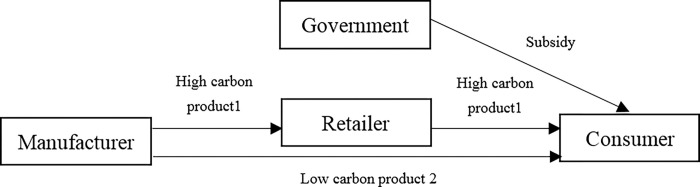
Dual channel supply chain under carbon subsidy mode.

If the government cancels the carbon tax on manufacturer and provides subsidy to consumers who purchase low carbon product 2, the dual channel supply chain under low carbon subsidy mode is shown in Fig 2.

The relationship between product demands and retail prices of high carbon product 1 and low carbon product 2 is consistent with 4.1, $q_{1}=a_{1}-b_{1}p_{1}+c(p_{2}-s),q_{2}=a_{2}-b_{2}(p_{2}-s)+cp_{1}$. Retailer profit is the same as (3), $\pi_{r}^{2}=q_{1}p_{1}-q_{1}w_{1}$. Since the government does not impose carbon tax on manufacturer producing high carbon product, the profit expression for manufacturer is:

$$Max\;\pi_{f}^{2}=q_{1}(w_{1}-v_{1})+q_{2}(p_{2}-v_{2})$$ (16)

The government’s decision making goal of social welfare is the largest, and there is no carbon tax revenue.


$$Max\;\pi_{g}^{2}=\psi^{2}+\pi_{r}^{2}+\pi_{f}^{2}-q_{2}s-\sum_{i=1}^{2}q_{i}m_{i}$$ (17)

Through similar solution to 4.1, we can get the optimal value of social welfare $\pi_{g}^{2}$:

$$s^{2*}=\frac{c(ah+c\varepsilon v_{1}+4cm_{2})+b_{1}[4a(1-h)-(4\varepsilon b_{2}-3c)v_{1}-(8b_{2}-4cr)m_{2}]}{4b_{1}b_{2}-3c^{2}}$$ (18)

The results for $p_{1}^{2*},p_{1}^{2*},q_{1}^{2*},q_{2}^{2*}$ and $w_{1}^{2*}$ are very complex, and the specific results are shown in Table 2.

### 4.3 Decision of dual channel supply chain under carbon tax mode

If the government cancels the subsidy for consumers who buy low carbon product 2 and only imposes carbon tax on manufacturer, consumers can not directly enjoy the carbon subsidy, and the relationships between demands and retail prices of high carbon product 1 and low carbon product 2 are expressed as follows:

$$q_{1}=a_{1}-b_{1}p_{1}+cp_{2}$$ (19)

$$q_{2}=a_{2}+cp_{1}-b_{2}p_{2}$$ (20)

**Fig 3:**
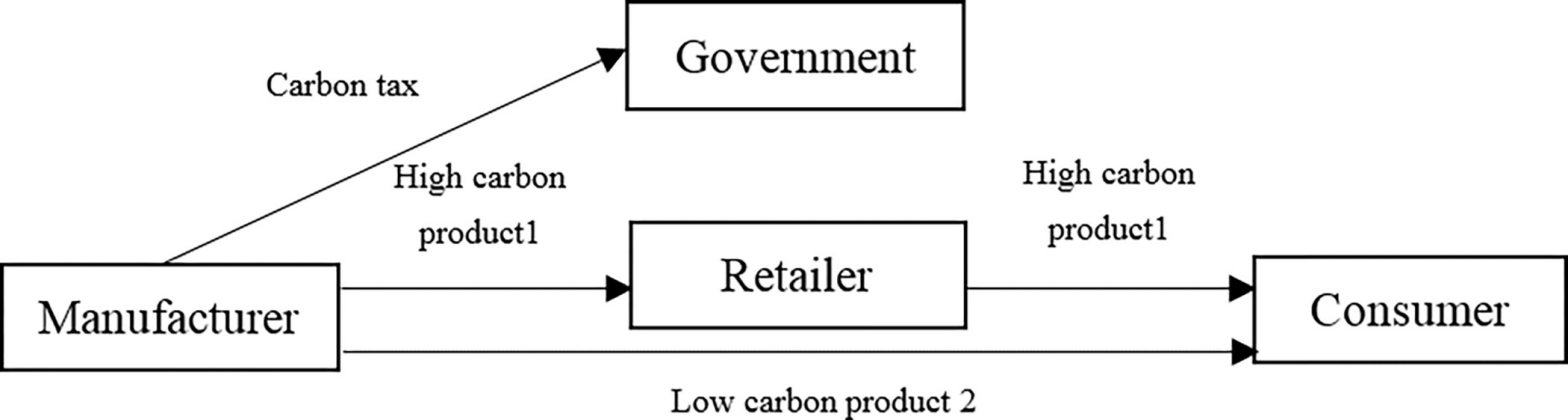
Dual channel supply chain under carbon tax mode.

The dual channel supply chain under carbon tax mode is shown in Fig 3.

In the case where only the government imposes carbon tax on the manufacturer, the retailer profit $\pi_{r}^{3}$ and manufacturer profit $\pi_{f}^{3}$ is the same as 4.1, $\pi_{r}^{3}=q_{1}p_{1}-q_{1}w_{1},\pi_{f}^{3}=q_{1}(w_{1}-v_{1})+q_{2}(p_{2}-v_{2})$. Since the carbon subsidy is cancelled, the government does not need to consider the government expenditure in the form of subsidy to consumers. Therefore, the government objective, i.e. social welfare function is expressed as:

$$\pi_{g}^{3}=\psi^{3}+\pi_{r}^{3}+\pi_{f}^{3}+q_{1}\tau -\sum_{i=1}^{2}q_{i}m_{i}$$ (21)

It is easy to prove that $\pi_{g}^{3}$ is a concave function of *τ*. Through the similar solving process to 4.1, we can acquire the optimal solution of social welfare $\pi_{g}^{3}$, which means the carbon tax to produce unit high carbon product 1 is:

$$\tau^{3*}=\frac{3(b_{1}-c\varepsilon )v_{1}-3ah-4(c-rb_{1})m_{2}}{b_{1}}$$ (22)

The optimal wholesale price of manufacturer toward high carbon product 1 under carbon tax is:

$$w_{1}^{3*}=\frac{\begin{array}{c} c^{2}(3ah+3c\varepsilon v_{1}+4cm_{2})+4b_{1}^{2}b_{2}(v_{1}+rm_{2})-\\ b_{1}\{b_{2}(2ah+3c\varepsilon v_{1}+4cm_{2})+c[a(h-1)+4cv_{1}+4crm_{2}]\} \end{array}}{2b_{1}(b_{1}b_{2}-c^{2})}$$ (23)

The optimal retail prices of high carbon product 1 and low carbon product 2 are:

$$p_{1}^{3*}=\frac{c^{2}(ah+c\varepsilon v_{1}+2cm_{2})+2b_{1}^{2}b_{2}(v_{1}+rm_{2})-cb_{1}[a(h-1)+(2c+\varepsilon b_{2})v_{1}+2(cr+b_{2})m_{2}]}{2b_{1}(b_{1}b_{2}-c^{2})}$$ (24)

$$p_{2}^{3*}=\frac{c(ah-c\varepsilon v_{1})+b_{1}[a(1-h)+\varepsilon b_{2}v_{1}]}{2(b_{1}b_{2}-c^{2})}$$ (25)

The demands of high carbon product 1 and low carbon product 2 are:

$$q_{1}^{3*}=ah+v_{1}(c\varepsilon -b_{1})+m_{2}(c-rb_{1})$$ (26)

$$q_{2}^{3*}=\frac{b_{1}[a(1-h)+(2c-\varepsilon b_{2})v_{1}+2crm_{2}]-c(ah+c\varepsilon v_{1}+2cm_{2})}{2b_{1}}$$ (27)

### 4.4 Comparison of three models

Based on the above calculations, we can obtain the optimal solutions and objectives for the three models (Table 2).

The specific expressions for *A*1, *A*2, *A*3, *B*1, *B*2, *B*3, D, *E*, *F*, *G*, *H*1, *H*2, *K*, *L* are shown in the S1 Appendix.

Through the Table 2, we can see that, obviously, $q_{1}^{3*}=q_{1}^{1*}$, which means that the demand of high carbon product 1 under carbon tax + subsidy mode is the same as that under the carbon tax mode. This indicates that when the government cancels the subsidy to consumers who buy low carbon product 2, only the demand of low carbon product 2 will be affected, while the demand of high carbon product 1 will not be affected.

**Theorem 5:** $\pi_{g}^{1}>max(\pi_{g}^{2},\pi_{g}^{3})$.

**Prove**: Because:

$$\pi_{g}^{1}-\pi_{g}^{2}=\frac{{\left\{2c^{2}(ah+c\varepsilon v_{1}+cm_{2})+b_{1}^{2}b_{2}(3v_{1}+4rm_{2})-b_{1}[b_{2}(3ah+2c\varepsilon v_{1}+2cm_{2})+c(a-ah+3cv_{1}+4crm_{2})]\right\}}^{2}}{8b_{1}(b_{1}b_{2}-c^{2})(4b_{1}b_{2}-3c^{2})}>0$$ (28)

$$\pi_{g}^{1}-\pi_{g}^{3}=\frac{{\left\{c(ah+c\varepsilon v_{1}+2cm_{2})+b_{1}[a(1-h)-b_{2}(\varepsilon v_{1}+2m_{2})]\right\}}^{2}}{8b_{1}(b_{1}b_{2}-c^{2})}>0$$ (29)

we can get $\pi_{g}^{1}>max(\pi_{g}^{2},\pi_{g}^{3})$.

According to theorem 5, compared to modes with only subsidy or carbon tax, social welfare under subsidy + carbon tax mode is the maximal. It is obvious that if the government wants to achieve maximum social welfare, it needs to implement both carbon tax and subsidy policies.

**Theorem 6:** $\pi_{r}^{1}=\pi_{r}^{3}$.

**Prove**: Because $q_{1}^{1*}=q_{1}^{3*}$, it can be proved through calculation that $\pi_{r}^{1}=\pi_{r}^{3}=\frac{{\left[ah+(c\varepsilon -b_{1})v_{1}+(c-rb_{1})m_{2}\right]}^{2}}{b_{1}}$.

Through theorem 6 we know that the retailer profit under subsidy + carbon tax mode is the same as that under carbon tax mode, and that the demand of high carbon product 1 will not change neither. The government imposes carbon tax on manufacturer to produce high carbon product 1, which will lead to an increase in the wholesale price of high carbon product 1 ($\frac{Dw_{1}^{1*}}{D\tau }=\frac{b_{1}b_{2}+c^{2}}{2(b_{1}b_{2}-c^{2})}>0$). However, the retailer will transfer the increase portion to consumers. It can be seen that the implementation of carbon tax policy by the government will not have an impact on the retailer profit in traditional channel.

## 5. Numerical analysis

This section aims to test the conclusion of model deduction of the previous section through numerical calculation, and further analyzes the social welfare, manufacturer profit, retailer profit and other relevant parameters of the dual channel low carbon supply chain considering product substitution under the three modes of subsidy + carbon tax, subsidy only and carbon tax only. Since it is difficult to obtain actual data, this section makes relevant calculations through hypothetical and general data [42].

Setting *a* = 380, *b*_1_ = 11, *b*_2_ = 9, *c* = 5.3, *v*_1_ = 3.8$, *m*_2_ = 0.4$, ε = 7.5, *r* = 60, *h* = 0.5, the specific parameter settings are as Table 3:

**Table 3:** Value of parameters under three modes.

Mode Parameters	Subsidy + carbon tax	Subsidy	Carbon tax
*π*_*g*_($)	767.11	753.11	600.60
*π*_*f*_($)	1661.12	2061.24	625.03
*π*_*r*_($)	126.96	266.15	126.96
*q*_1_	37.37	54.11	37.37
*q*_2_	77.24	73.21	30.91
*w*_1_($)	24.40	21.06	27.87
*p*_1_($)	27.80	25.98	31.26
*p*_2_($)	43.28	43.90	36.09
*τ*($)	6.69	-	13.62
*s*($)	14.38	15.63	-

It is shown in Table 3 that: social welfare is the largest under the subsidy + carbon tax mode, followed by the subsidy mode and the minimum under the carbon tax mode, verified the correctness of theorem 5. The manufacturer profit is the largest in the subsidy mode and the carbon tax mode is the smallest. However, the profit of retailer is the largest under the subsidy mode, followed by the subsidy + carbon tax mode, and the carbon tax mode is the smallest. The demand of high carbon product 1 is the largest under the subsidy mode, and the subsidy + carbon tax mode is equal to the carbon tax mode. The demand for low carbon product 2 is the largest under the subsidy + carbon tax mode, and the carbon tax mode is the smallest. The wholesale price and retail price of high carbon product 1 are the highest under the carbon tax mode, and the retail price of low carbon product 2 is the highest under the subsidy mode.

### 5.1 Sensitivity analysis

The main differences between low carbon product 2 and high carbon product 1 are reflected in environmental hazard and product cost, so we analyze the sensitivity of ε and *r*. The influence of *ε* on all the parameters under three modes are shown in Table 4:

**Table 4:** The influence of *ε* on all the parameters under three modes.

Parameters	*ε*	Subsidy + carbon tax	Subsidy	Carbon tax
*π*_*g*_	7.2	861.01	842.04	677.04
	7.5	767.11	753.11	600.60
	7.8	687.90	675.13	543.75
*π*_*f*_	7.2	1811.24	2255.12	607.42
	7.5	1661.12	2061.24	625.03
	7.8	1541.13	1891.21	660.11
*π*_*r*_	7.2	89.22	234.73	89.22
	7.5	126.96	266.15	126.96
	7.8	171.33	299.54	171.33
*q*_1_	7.2	31.33	50.81	31.33
	7.5	37.37	54.11	37.37
	7.8	43.41	57.40	43.41
*q*_2_	7.2	87.50	82.81	37.49
	7.5	77.24	73.21	30.91
	7.8	66.98	63.61	24.32
*w*_1_	7.2	24.95	21.06	28.69
	7.5	24.40	21.06	27.87
	7.8	23.85	21.06	27.04
*p*_1_	7.2	27.80	25.68	31.54
	7.5	27.80	25.98	31.26
	7.8	27.80	26.28	30.99
*p*_2_	7.2	43.28	44.00	35.52
	7.5	43.28	43.90	36.09
	7.8	43.28	43.80	36.66
*τ*	7.2	7.79	-	15.26
	7.5	6.69	-	13.62
	7.8	5.59	-	11.97
*s*	7.2	15.52	16.97	-
	7.5	14.38	15.63	-
	7.8	13.24	14.28	-

The increase of ε indicates the wholesale price increase of low carbon product 4. According to Table 3, with the increase of *ε*, under subsidy + carbon tax mode *π*_*r*_ and *q*_1_ will increase and the following parameters will decrease: *π*_*g*_, *π*_*f*_, *q*_2_, *w*_1_, *τ*, *s*, with no influence on *p*_1_ and *p*_2_. Under subsidy mode, *π*_*r*_, *q*_1_, and *p*_1_ will increase, *π*_*g*_, *π*_*f*_, *q*_2_, *p*_2_, and *s* will decrease, and *w*_1_ will not change. Under carbon tax mode, *π*_*f*_, *π*_*r*_, *q*_1_, and *p*_2_ will increase while *π*_*g*_, *q*_2_, *w*_1_, *p*_1_ and *τ* will decrease. It can be deducted that the increase of the cost of low carbon product 2 will lead to the rise of retailer profit and sales of high carbon product 1, while the government target and sales of low carbon product 2 will decrease. Because the increase of producing cost of low carbon product 2 causes the decrease of its demand, it will directly result in the decrease of retailer profit and government target. Due to the competitive relationship between high carbon product 1 and low carbon product 2, the sales of high carbon product 1 will increase.

The influence of *r* on all the parameters under three modes are shown in Table 5:

**Table 5:** The influence of *r* on all the parameters under three modes.

Parameters	*r*	Subsidy + carbon tax	Subsidy	Carbon tax
*π*_*g*_	58	800.52	796.72	634.01
	60	767.11	753.11	600.60
	62	740.73	710.14	574.22
*π*_*f*_	58	1794.84	2018.12	758.70
	60	1661.12	2061.24	625.03
	62	1555.67	2105.74	519.53
*π*_*r*_	58	193.79	274.79	193.79
	60	126.96	266.15	126.96
	62	74.20	258.40	74.20
*q*_1_	58	46.17	54.90	46.17
	60	37.37	54.11	37.37
	62	28.57	53.31	28.57
*q*_2_	58	73.00	70.90	26.67
	60	77.24	73.21	30.91
	62	81.48	75.52	35.15
*w*_1_	58	24.95	21.06	26.67
	60	24.40	21.06	27.87
	62	26.00	21.06	29.47
*p*_1_	58	27.00	26.05	30.46
	60	27.80	25.98	31.26
	62	28.60	25.90	32.06
*p*_2_	58	43.28	43.60	36.09
	60	43.28	43.90	36.09
	62	43.28	44.20	36.09
*τ*	58	3.49		10.42
	60	6.69	-	13.62
	62	9.89		16.82
*s*	58	14.38	15.03	
	60	14.38	15.63	-
	62	14.38	16.23	

The bigger the value of *r* is, the larger environmental hazard high carbon product 1 causes, and contrarily the environmental hazard created by low carbon product 2 is smaller. Under subsidy + carbon tax mode, *q*_2_, *w*_1_, *p*_1_, and *τ* will increase, *π*_*g*_, *π*_*f*_, *π*_*r*_, and *q*_1_ will decrease, verified the correctness of Theorem 4, and *p*_2_ and *s* receive no influence. Under subsidy mode, *π*_*f*_, *q*_2_, *p*_2_, and *s* will increase, *π*_*g*_, *π*_*r*_, *q*_1_, and *p*_1_ will decrease, and *w*_1_ doesn’t change. Under carbon tax mode, *q*_2_, *w*_1_, *p*_1_, and *τ* will increase, *π*_*g*_, *π*_*f*_, *π*_*r*_, and *q*_1_ will decrease, and *p*_2_ will stay unchanged. We can see that the increase of *r* causes the rise of sales of low carbon product 2 under all three modes as well as the decrease of manufacturer profit, government target and sales of high carbon product 1. Because of the increase of pollution from high carbon product 1, government will increase the tax levied on high carbon product 1, thus leading to smaller demand of products 1, then the decrease of manufacturer profit and government target.

### 5.2 Dual channel analysis

The manufacturer profit is the sum of the profit from selling high carbon product 1 through traditional channel and the profit from directly channel low carbon product 2. The main indicators that affect these two dual channels are the production cost of the low carbon product 2 and consumer product preferences. Therefore, this section mainly analyzes the impact of *ε* and *h* on manufacturer profit. Set *tradition* as profit from high carbon product 2 through traditional channel, *direct* as profit from low carbon product 1 through direct channel.

**Fig 4:**
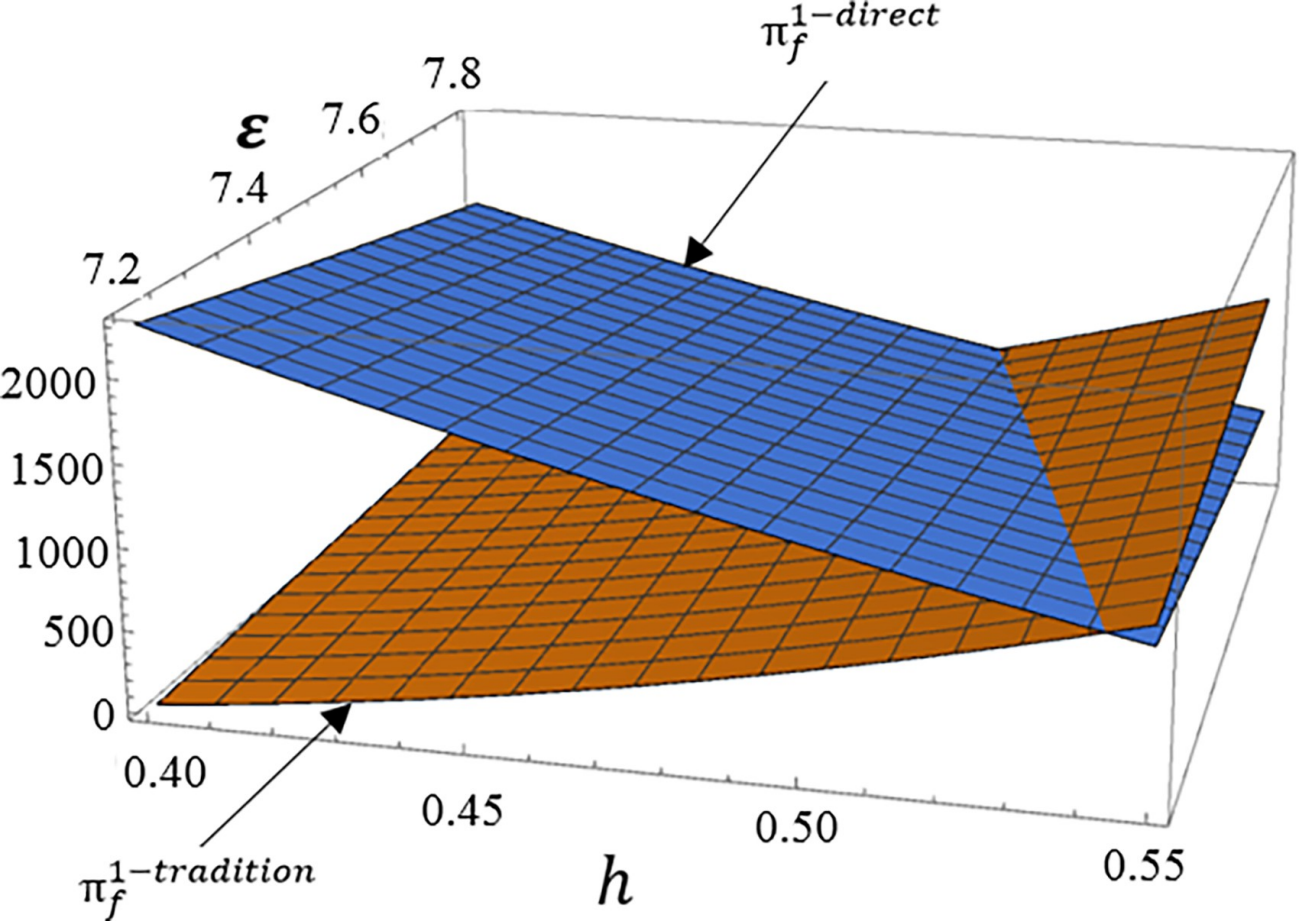
The impact of *ε* and *h* on dual channel supply chain under carbon tax + subsidy mode.

**Fig 5:**
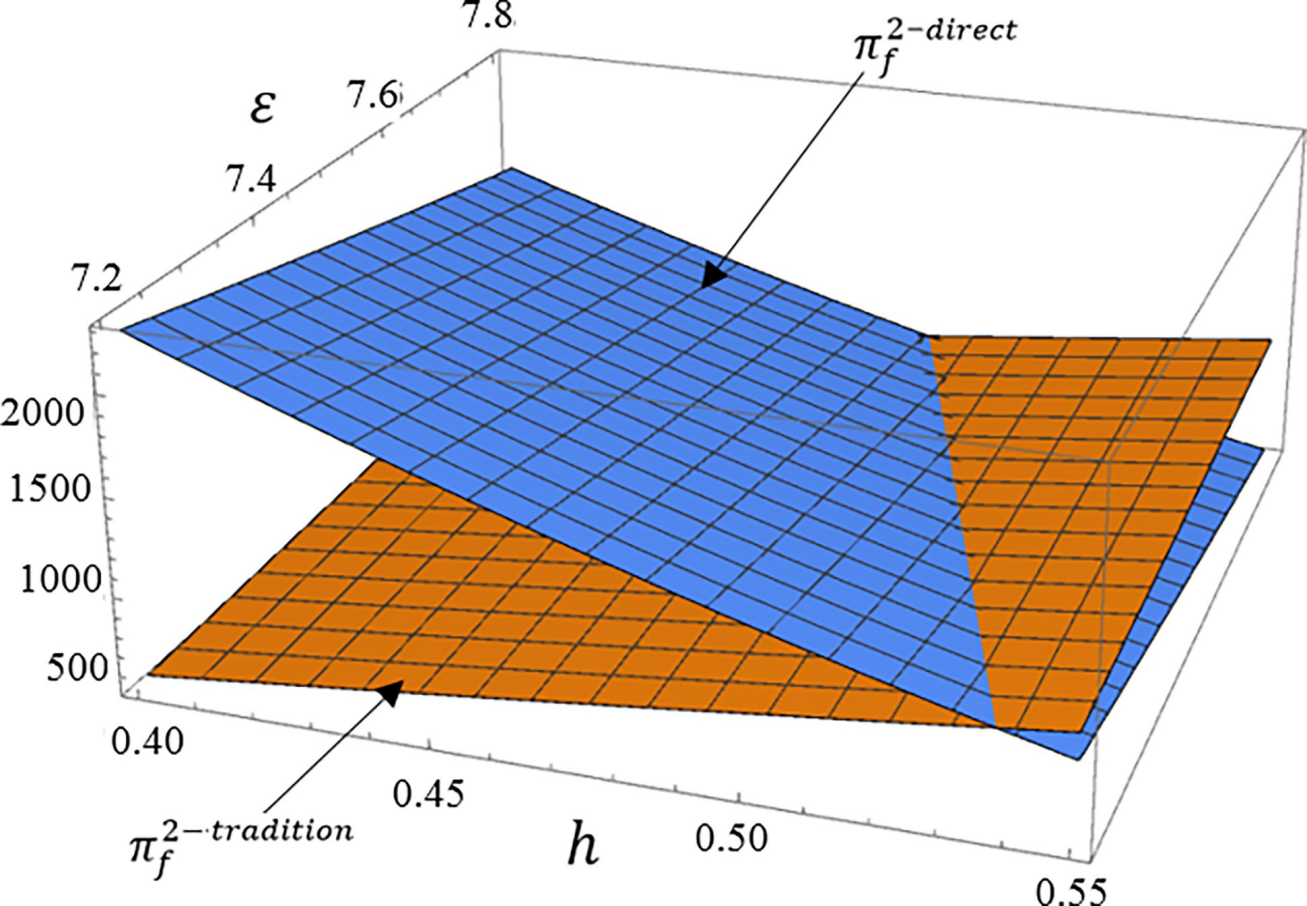
The impact of *ε* and *h* on dual channel supply chain under subsidy mode.

**Fig 6:**
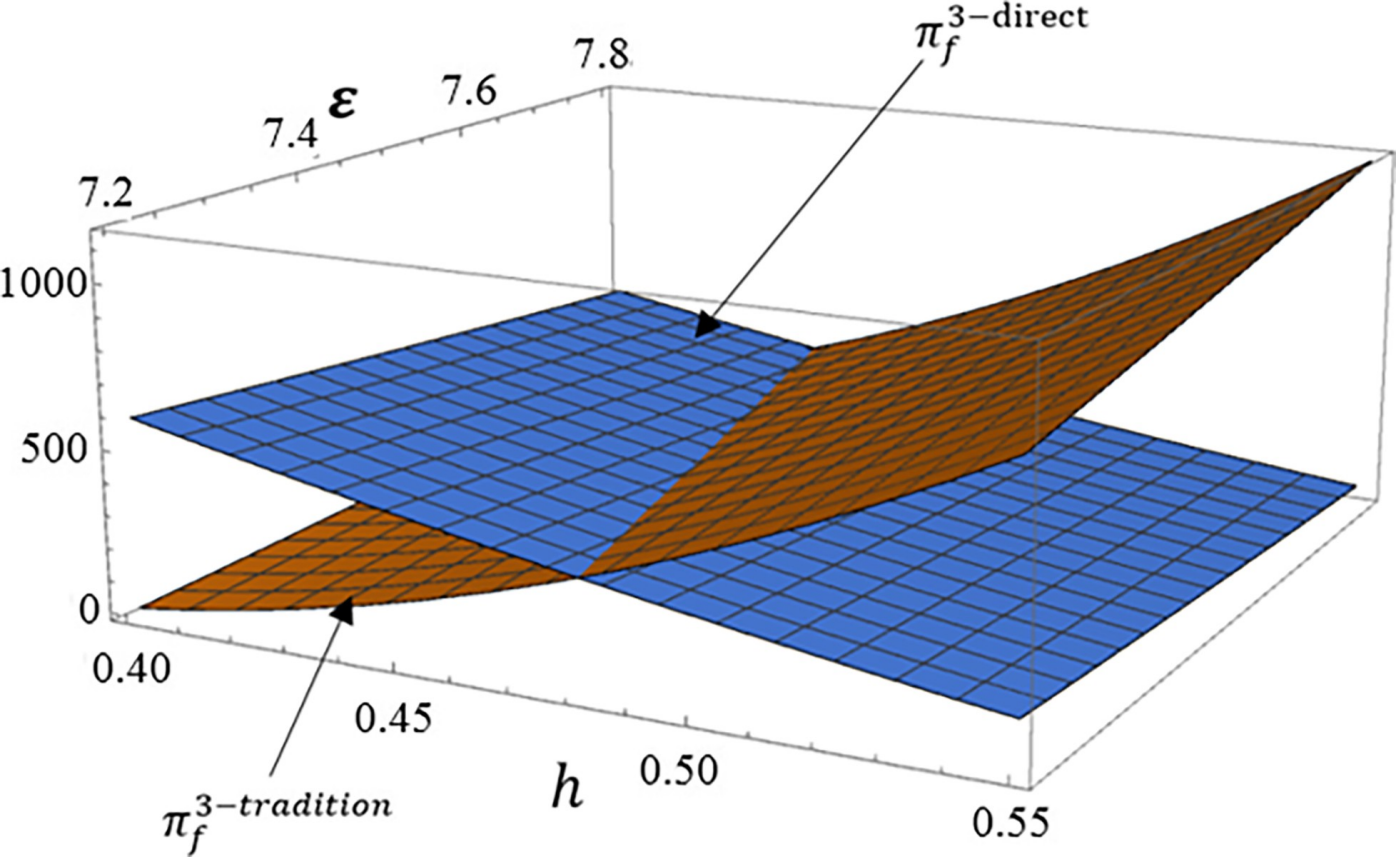
The impact of *ε* and *h* on dual channel supply chain under carbon tax mode.

According to Figs 4, 5, 6, we can see that as the proportion of consumers who prefer high carbon products in the total market *h* increases, the profit brought to manufacturer by traditional channel for high carbon product 1 will significantly increase, while the profit brought to manufacturer by direct channel for low carbon products 2 reduce. And the increase in product cost of low carbon product 2 will also bring the same result. Therefore, in order to enhance the competitive advantage of low carbon product 2, manufacturer need to focus on the consumer preference for low carbon product 2.

## 6. Conclusion

The core issue of supply chain management is product management. It needs a new sales channel for new product replacing old product in the market, and the dual channel provides the possibility of establishing the new sales channel. The purpose of product management is to meet consumer needs. With the strengthening of consumers’ awareness of environmental protection, low carbon products are becoming increasingly popular in the market, and government subsidy policy is further promoting consumers’ preference for low carbon products. Therefore, how to coordinate government, manufacturer, and retailer coordinate with each other to achieve maximum social performance, environmental performance, and economic benefits is a problem that needs to be solved. This research studies the impact of government carbon tax and low carbon subsidy on dual channel low carbon supply chain. We have built a two level dual channel supply chain, including a manufacturer and a retailer, in which the manufacturer produces both high carbon product through traditional channel and low carbon product through direct channel. The government subsidizes consumers who buy low carbon product and imposes carbon tax on manufacturers who produce high carbon product. We compare parameters such as the social welfare, manufacturer profit and retailer profit under the three modes of subsidy + carbon tax, subsidy only and carbon tax only. The purpose of this study is to provide effective suggestions for decision making among the government, manufacturer, and retailer, in order to achieve maximum social welfare, manufacturer profit, and retailer profit.

The findings of this study are as follows:

1. For social welfare, carbon tax + subsidy model is higher than subsidy model and carbon tax model, and is inversely proportional to the environmental hazard caused by high carbon product.
2. For manufacturer profit, subsidy model is the highest, followed by subsidy + carbon tax model, which is inversely proportional to the environmental hazard caused by high carbon product.
3. For retailer profit, subsidy + carbon tax model is equal to carbon tax model, which is inversely proportional to the environmental hazard caused by high carbon products.
4. The increase in the cost of low carbon product will lead to the increase in the profit of retailer and the sales of high carbon product under all three modes, and the decrease in the government target and the sales of low carbon product. The increase of the environmental hazard caused by high carbon product will lead to an increase in the sales of low carbon product under all three modes, as well as a decrease in the profit of manufacturer, government target and sales of high carbon product.
5. The increase of the market share of high carbon product or the product cost of low carbon product, will lead to increase the traditional channel profit and decrease the direct channel profit under the three modes.

However, the research still has certain limitations. For example, only two products are considered, but in reality, there are always many products that have substitution relations. At the same time, there is only one retailer and one manufacturer in this model, while multiple manufacturers and retailers are more common. Future research can focus on the following aspects that can be expanded: on the one hand, we hope to study the substitution of multiple products inside a dual-channel low-carbon supply chain, and to compare the impact of the government’s carbon tax and subsidy policies on this supply chain; on the other hand, we are looking forward to considering a more complex dual channel low carbon supply chain system with multiple manufacturers and retailers, and analyze the impact of the government’s carbon tax and subsidy policies on the decisions and profits of manufacturers and retailers.

## Supporting information

S1 Appendix (DOCX)
